# Reduction of Cell Proliferation by Acute C_2_H_6_O Exposure

**DOI:** 10.3390/cancers13194999

**Published:** 2021-10-05

**Authors:** Silvia Baldari, Isabella Manni, Giuliana Di Rocco, Francesca Paolini, Belinda Palermo, Giulia Piaggio, Gabriele Toietta

**Affiliations:** 1 Tumor Immunology and Immunotherapy Unit, IRCCS-Regina Elena National Cancer Institute, 00144 Rome, Italy; silvia.baldari@ifo.gov.it (S.B.); francesca.paolini@ifo.gov.it (F.P.); belinda.palermo@ifo.gov.it (B.P.); 2 Stabilimento Allevatore Fornitore Utilizzatore (SAFU), IRCCS-Regina Elena National Cancer Institute, 00144 Rome, Italy; isabella.manni@ifo.gov.it (I.M.); giulia.piaggio@ifo.gov.it (G.P.); 3 Unit of Cellular Networks and Molecular Therapeutic Targets, IRCCS-Regina Elena National Cancer Institute, 00144 Rome, Italy; giuliana.dirocco@ifo.gov.it

**Keywords:** cell proliferation, alcohol, bone marrow, binge drinking, in vivo bioluminescence imaging, ethanol, acetaldehyde, alcoholism, animal model, nuclear factor Y

## Abstract

**Simple Summary:**

Alcoholic beverages and acetaldehyde formed during their metabolism are carcinogenic to humans. Alcohol drinking may affect bone marrow stem cell niche, suppressing physiological hematopoiesis and ultimately reducing the organism’s capacity to fight against cancer, infections, and to promote tissue regeneration. To elucidate in vivo the cellular mechanisms associated with alcohol intake toxicity, we used a mouse model in which proliferating cells produce the firefly’s light-emitting protein. In this animal, alcohol exposure transiently “turns off the light”, indicating a negative effect on cell proliferation in the bone marrow and spleen. Pharmacological treatment with substances interfering with ethanol metabolism, reducing acetaldehyde production, partially restores the physiological cell proliferation rate. Over 560 million people worldwide have increased susceptibility to acetaldehyde toxicity and 4% of cancer deaths are attributable to alcohol. Our model might provide a suitable tool to further investigate in vivo the effects of alcohol metabolism and aldehydes production on carcinogenesis.

**Abstract:**

Endogenous acetaldehyde production from the metabolism of ingested alcohol exposes hematopoietic progenitor cells to increased genotoxic risk. To develop possible therapeutic strategies to prevent or reverse alcohol abuse effects, it would be critical to determine the temporal progression of acute ethanol toxicity on progenitor cell numbers and proliferative status. We followed the variation of the cell proliferation rate in bone marrow and spleen in response to acute ethanol intoxication in the MITO-Luc mouse, in which NF-Y-dependent cell proliferation can be assessed in vivo by non-invasive bioluminescent imaging. One week after ethanol administration, bioluminescent signals in bone marrow and spleen decreased below the level corresponding to physiological proliferation, and they progressively resumed to pre-treatment values in approximately 4 weeks. Boosting acetaldehyde catabolism by administration of an aldehyde dehydrogenase activity activator or administration of polyphenols with antioxidant activity partially restored bone marrow cells’ physiological proliferation. These results indicate that in this mouse model, bioluminescent alteration reflects the reduction of the physiological proliferation rate of bone marrow progenitor cells due to the toxic effect of aldehydes generated by alcohol oxidation. In summary, this study presents a novel view of the impact of acute alcohol intake on bone marrow cell proliferation in vivo.

## 1. Introduction

Alcoholic beverages contain different amounts of ethanol (ethyl alcohol, CH_3_−CH_2_−OH or C_2_H_6_O) and low levels of methanol (methyl alcohol, CH_3_-OH, or CH_4_O). Acetaldehyde and formaldehyde are endogenously produced, respectively, during the metabolism of ethanol and methanol ingested via alcoholic drinks. The International Agency for Research on Cancer, part of the World Health Organization, has classified alcoholic beverages, acetaldehyde, and formaldehyde as “carcinogenic to humans” (Group 1 Carcinogen) [1].

Consumption of alcoholic beverages may reduce the generation of inflammatory mediators leading to immunologic alterations, with increased susceptibility to infection and tumor formation and reduced healing after traumatic injury [2,3,4,5,6,7,8]. While the fact that chronic alcohol abuse severely damages immune function is well-established, only recently the role of acute ethanol exposure (binge drinking) in deteriorating protective immunity has been investigated [9]. Alterations in the hematologic profile have been observed not only in alcoholics, but also in short-term moderate alcohol drinkers [10]. In addition, maternal ethanol consumption causes immune deficiencies in newborns [11]. Some of these harmful consequences of alcohol exposure on the hematopoietic system [12] may determine an increased risk of some types of cancer [13,14].

Alcohol bone marrow toxicity leads to a reduction in blood cells precursors, thus interfering with blood cell production and functionality [15]. As a result, alcoholic patients may experience anemia, bleeding disorders, and, as mentioned above, compromised immune response [16]. Hematopoiesis is a highly regulated process by which blood and immune cells are replaced and produced by a small population of hematopoietic stem cells (HSCs). Some of alcohol’s adverse effects are indirect, due to nutritional deficiencies that impair the production and function of various blood cells. Direct effects of alcohol toxicity target the bone marrow (BM), possibly lowering the number of blood cell precursors [15]. In fact, the reduction of red blood cells (RBCs), white blood cells (WBCs), and platelets suggests that ethanol toxicity may target BM precursor cells [17]. In line with this hypothesis, the formation of vacuoles, which interfere with cells’ functionality, has been observed in RBC and WBC precursors. Hematopoiesis also involves the interaction of the developing cells with BM stroma, which provides the appropriate microenvironment for differentiation [18]. Ethanol could therefore affect the BM progenitor cells directly and/or act indirectly by altering the HSC microenvironment. As a matter of fact, alcohol has a harmful effect on BM stromal cells, which has been associated with the reduction of bone mass and decreased bone formation observed in alcoholics [19]. Moreover, several studies have suggested a direct toxic effect of ethanol and/or its metabolites on both neuronal [20] and hematopoietic progenitor cells [21]; however, the exact mechanisms need to be further characterized and probably depend on the target organ [22].

Some studies on the toxicity of ethanol and its derivatives have been performed on cultured precursor cells of different origin (bone marrow- and adipose tissue-derived stromal cells, neuronal precursors, hematopoietic cells) [20,23,24]. These studies are only partially informative since in vitro cells are exposed to rather elevated and constant doses of ethanol. Therefore, in vivo studies are needed to more precisely define the physiological and pathological processes related to alcohol intoxication. Performing studies of acute ethanol exposure in humans raises serious ethical concerns. Recently, studies on non-human primates have been performed, demonstrating that alcohol intake may result in impairment of the bone marrow niche and hematopoietic stem/progenitor function [25]. Notwithstanding the complexity and limitations of alcohol drinking studies in rodents, the existence of transgenic models facilitates mechanistic studies [26].

In the current work, we explored in vivo the effects of experimental acute ethanol intoxication in the transgenic MITO-Luc mouse [27]. In this animal model, luciferase activity is under the control of the nuclear factor-Y-dependent cyclin B2 (NF-Y) promoter and consequently restricted to proliferating cells. Consequently, in this transgenic mouse, it is possible to visualize and map by in vivo bioluminescence imaging (BLI) the regions of active cell proliferation. In particular, in MITO-Luc mice, high luciferase activity can be detected in spleen, testis, and bone marrow (vertebral column, sternum, femur), while non-proliferating tissues, such as lung, brain, heart, aorta, skeletal muscle, liver, and kidney, do not emit light under physiological conditions [27]. This experimental model has been instrumental in studies analyzing cell proliferation in response to hyperbilirubinemia [28], toxic insult [29], and ischemic [30] and spinal cord [31] injuries.

## 2. Materials and Methods

### 2.1. Experimental Animal Procedures

Animals used in the study were 6–8 weeks old male and female albino MITO-Luc mice [27] gathered in groups of 4 to 8 animals each. Gavage administration of a solution of ethanol (dose range 3–6 g/kg body weight) in 0.9% saline in a total volume of 150 μl-solution per 10 g of body weight was performed. According to previous reports, this dose range corresponds to a binge-like drinking pattern in humans [32]. Randomly selected animals received intra-peritoneal (i.p.) administration of the ALDH activator ALDA-1 (5 mg/kg) [33] or the ALDH inhibitor cyanamide (25 mg/kg) [34] (Sigma Aldrich, St. Louis, MO, USA) 30 min before ethanol treatment. Similarly, a group of animals received i.p. a solution containing polyphenols (50 mg/kg) (Phenolea Active Complex, kindly provided by Phenofarm, Rieti, Italy) [35] 2 h before alcohol administration. Methanol was administered (1.5 g/kg body weight) in 0.9% saline solution in a total volume of 150 μL per 10 g of body weight [36]. Experimental procedures for assessment of cell proliferation in the MITO-Luc mouse model conformed to Animal Care guidelines (D.lgs 26/2014, 4 March 2014).

### 2.2. In Vivo and Ex Vivo Optical Bioluminescence Imaging

BLI analysis was performed using the IVIS Lumina II equipped with the Living Image 4.7.3 software for data quantification (PerkinElmer, Waltham, MA, USA), as previously described [37]. For in vivo imaging, mice were anesthetized, and D-luciferin (150 mg/kg body weight) (PerkinElmer) dissolved in phosphate-buffered saline (PBS) was administered i.p. 10 min before analysis. For ex vivo imaging, animals were euthanized, organs excised, placed into tissue culture dishes, and incubated for 10 min in PBS containing D-luciferin (150 µg/mL) before analysis [38].

### 2.3. Complete Blood Counts

Blood was collected from the jugular vein and transferred into heparin-containing vials. Complete blood counts were performed automatically on a hematology analyzer.

### 2.4. Proteome Analysis

A specific Proteome Profiler™ Mouse Cytokine Array (R&D Systems, Minneapolis, MN, USA) was used according to the manufacturer’s specifications to determine plasmatic levels of a panel of 40 soluble factors including G-CSF, M-CSF, GM-CSF, SCF, IL-3, IL-6, TNFα, and ICAM-1 in response to acute alcohol exposure compared to control samples.

### 2.5. Immunoblot Analysis

Spleen homogenates (100 μg) were separated by sodium dodecyl sulfate–polyacrylamide gel electrophoresis under reducing conditions and transferred into nitrocellulose membranes. Immunoblotting was performed following standard protocols using primary antibodies against SDF-1 (Santa Cruz Biotechnology, Dallas, TX, USA), SOD2 (Upstate Biotechnology–Millipore, Temecula, CA, USA) [37], BAX (Santa Cruz Biotechnology), and IκBα (Cell Signaling Technology, Danvers, MA, USA). Glyceraldehyde phosphate dehydrogenase (GAPDH) was used as a protein loading control. Immunoblot densitometry band quantification was performed using the ImageJ software (https://imagej.nih.gov/ij/) (accessed on 4 October 2021). All uncropped WB was shown in Figure S1.

### 2.6. Statistical Analysis

Results are expressed as means ± standard error of the mean (SEM). We used the INSTAT software (GraphPad, San Diego, CA, USA) for data analysis and comparisons between groups. The significance of differences was assessed with a two-tailed Student *t*-test for unpaired data; a *p*-value below 0.05 was considered statistically significant.

## 3. Results

### 3.1. Alcohol Intake Transiently Modulates Bioluminescence Signals Associated with Cell Proliferation in the MITO-Luc Mouse

**Figure 1:**
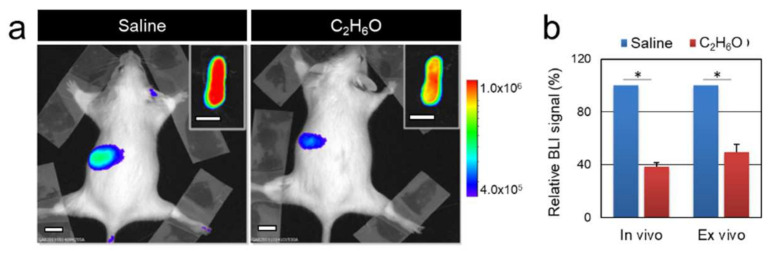
Response of NF-Y-dependent cell proliferation to acute ethanol administration in the MITO-Luc mouse model. (**a**) Bioluminescence imaging analysis performed on a representative animal 5 days after administration of saline or C_2_H_6_O solution. The insets show ex vivo imaging of excised spleens. (**b**) Regions of interests were drawn around spleens and quantified BLI intensities relative to saline control levels are shown in the graph. White scale bars: 1 cm. The pseudo-color scale bar represents the relative bioluminescent signal intensities from the lowest (blue) to the highest (red); radiance is expressed in photons per second per square centimeter per steradian (photons/s/cm^2^/sr). The asterisk (*) indicates a statistically significant difference between the indicated groups (*p* ≤ 0.05).

Luciferase (Luc) expression in the MITO-Luc mouse is restricted to proliferating cells, being under the control of the NF-Y transcription factor. Therefore, luciferase-mediated light emission can represent an easily detectable surrogate for assessing in vivo cell proliferation. We determined the systemic effect of acute alcohol administration on cell proliferation in MITO-Luc mice subjected to a single administration of ethanol solution (6 g/kg body weight), described as the dose mimicking binge drinking in humans [9,32]. Longitudinal BLI imaging was performed, beginning before treatment (baseline) and then at different time points after alcohol administration. Five days after acute ethanol administration, BLI signals in the spleen were below the level observed before ethanol administration and in control animals receiving saline solution, corresponding to physiological proliferation (Figure 1). Luciferase levels in liver samples remain unaltered, as assessed by in vivo and ex vivo (not shown) BLI analysis, confirming that acute alcohol exposure does not induce hepatic cell proliferation [39].

**Figure 2:**
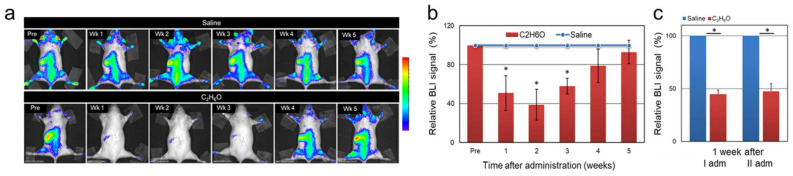
Kinetics of the response of NF-Y-dependent cell proliferation to acute ethanol administration in the MITO-Luc mouse model. Longitudinal BLI imaging performed at different time points after administration on MITO-Luc mice administered with either vehicle (saline) or ethanol solution. Luciferase emission was determined by BLI in (**a**) a representative animal and (**b**) in each acquisition, regions of interests countering the whole animal were delineated and BLI emission quantified using the Living Image software. The graph illustrates BLI signal intensities relative to saline control levels set as 100%. (**c**) MITO-Luc mice were administered with either vehicle or C_2_H_6_O solution; after 8 weeks of recovery, the administration was repeated at the same dose. Quantification of the BLI signals was performed 1 week after each administration The pseudo-color scale bar indicates the relative signal intensity from the lowest (blue) to the highest (red) (range 1 × 10^4^–1 × 10^5^ photons/s/cm^2^/sr). The asterisk (*) indicates a statistically significant difference compared with the relative saline group.

One week after the administration, a residual BLI signal was detectable in the spleen, while bioluminescence in the BM was below the detection limit. Suppression of BM and spleen cell proliferation was transient and progressively resumed to control values in approximately 4–5 weeks (Figure 2a,b). These results are consistent with previous reports describing hematopoietic impairment associated with alcohol-dependent direct and/or indirect damage of hematopoietic stem cells [40], recovered in approximately a month [41].

In addition, we performed complete blood counts 1 week after acute administration, in correspondence with the reduction of the BLI signal. We did not detect any significant difference in the total number of erythrocytes, leukocytes, and platelets between the saline and the ethanol-administered group.

Furthermore, in a similar fashion, we examined the effects of multiple binge-like episodes on cell proliferation. To this end, a group of 4 MITO-Luc mice was administered with an ethanol solution by gavage administration and BLI analysis was performed at different time-points throughout the experiment. Animals were then allowed 8 weeks to recover, then administration was repeated at the same dose. After the second episode of alcohol administration, we observed a transient reduction of bioluminescence similar in magnitude to that observed after the first alcohol intoxication episode (Figure 2c). This indicates that repeated cycles of binge-like ethanol drinking have a similar impact on NF-Y-dependent cell proliferation in hematopoietic tissues in the MITO-Luc mouse.

#### 3.1.1. Modulation of Aldehyde Dehydrogenases Activity Affects Ethanol-Induced Alteration of Bioluminescence

**Figure 3:**
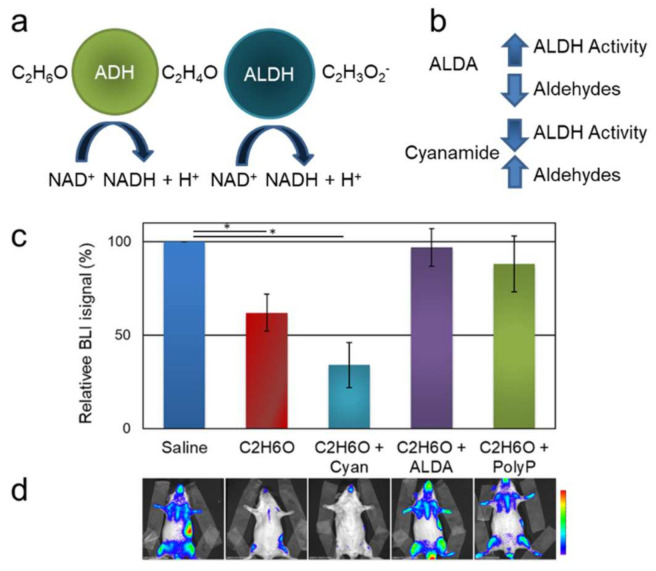
Effects of pharmacological treatments on NF-Y-dependent cell proliferation in MITO-Luc mice administered C_2_H_6_O. (**a**) Illustration of the metabolism of ethanol into acetaldehyde and acetate. ADH: alcohol dehydrogenase; ALDH: aldehyde dehydrogenase. (**b**) Schematic representation of the effects of pharmacological treatment with the ALDH inhibitor cyanamide or the activator ALDA. (**c**) Quantification of the BLI signals relative to the control (saline) group in animals administered C_2_H_6_O treated either with cyanamide, ALDA, or the antioxidant compound Phenolea Active Complex (PolyP). (**d**) Representative BLI analysis in one mouse per group at approximately 10 days after treatment. The pseudo-color scale bar indicates the relative signal intensity from the lowest (blue) to the highest (red) (range 1 × 10^4^–1 × 10^5^ photons/s/cm^2^/sr). The asterisk (*) indicates a statistically significant difference between the indicated groups (*p* ≤ 0.05).

Ethanol is removed from the body through oxidation. In particular, alcohol dehydrogenases (ADH) catalyze the oxidation of ethanol to acetaldehyde, known as a carcinogen and a key generator of free radicals [42], which is then converted to non-toxic acetate by aldehyde dehydrogenases (ALDH) (Figure 3a). In particular, ALDH2 is the most efficient of the 19 human ALDH isoforms in detoxifying ethanol-derived acetaldehyde. It has been unambiguously demonstrated that endogenous aldehydes, such as the ones produced during ethanol metabolism, are genotoxic in hematopoietic cells [43,44,45].

In wild-type C57B6 mice, traces of acetaldehyde in blood and brain can be assessed within minutes upon ethanol administration [46]. We evaluated whether pharmacological treatment with ALDH modulators [the inhibitor cyanamide (25–50 mg/kg) and the activator ALDA-1 (8.5 mg/kg) (Figure 3b)] might determine any possible modification in the cell proliferation pattern observed in the ethanol-treated MITO-Luc mice (Figure 3c). Indeed, upon alcohol administration in the MITO-Luc mice, treatment with the ALDH activity inhibitor determined a severe toxic effect resulting in 50% mortality within 2 days upon administration, while the surviving animals displayed a more severe modulation of BLI compared with the animal receiving alcohol solution only. These data suggest that increased levels of acetaldehyde subsequent to inhibition of ALDH activity may result in increased toxicity on BM cells. No deaths occurred in control animals administered with either ethanol or cyanamide alone. Conversely, administration of the ALDH activity activator ALDA-1 restored, at least in part, BM cells’ physiological proliferative profile as assessed by BLI, possibly by reducing toxicity due to boosted acetaldehyde catabolism (Figure 3c). Taken together, these data point to acetaldehyde as a possible mediator of an alcohol-driven reduction of BM cells’ proliferation and to ALDH2 as an actionable therapeutic target.

#### 3.1.2. Polyphenol Administration Partially Restores Bioluminescence upon Ethanol Exposure

Ethanol metabolism generates free radicals, altering the cellular redox status and leading to cellular damage [47,48,49]. Fetal tissues are particularly vulnerable to oxidative damage since the levels of enzymatic and non-enzymatic antioxidants are lower than in adults [50]. Accordingly, antioxidant strategies have been proposed to counteract the toxic actions of alcohol-mediated oxidative stress [51,52]. Recently, the administration of polyphenols derived from olive leaves has been described to attenuate damages associated with chronic alcohol abuse by reduction of reactive oxygen species (ROS) in the serum [35]. We therefore evaluated whether the administration of antioxidants may affect the possible perturbation of BLI in mice receiving acute alcohol intoxication. In this study, we used a polyphenol blend derived from a standardized olive pulp (*Olea europaea* L.). This product, named Phenolea Active Complex, has been extensively characterized before [35,53]. Indeed, the delivery of olive-derived polyphenols before alcohol intoxication attenuates the reduction of BM cell proliferation (Figure 3c,d), while the administration of polyphenols alone did not produce any significant effect (not shown). This result further supports the role of olive polyphenols in conferring protection against ethanol-induced oxidative stress [35,54].

### 3.2. Acute Methanol Administration Transiently Modulates Cell Proliferation in the MITO-Luc Mice

In the same way as acetaldehyde [43], formaldehyde also exerts cytotoxic effects on HSCs [55,56]. Formaldehyde exposure can occur from a variety of exogenous sources, such as tobacco and e-cigarette smoke, aspartame and furniture, textiles, and cosmetics containing formaldehyde-based products, such as glue, paint, and resins. Formaldehyde is endogenously produced by enzymatic oxidative demethylation reactions and during catalase- and alcohol dehydrogenase-mediated oxidation of methanol contaminations ingested in alcoholic beverages [57].

**Figure 4:**
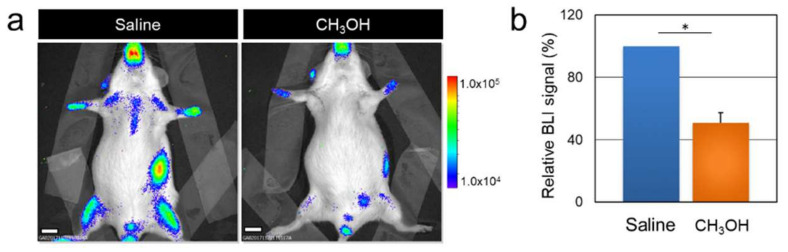
Effect of methanol administration on bioluminescent signal in MITO-Luc mice. Animals were administered either vehicle (saline) or methanol solution (CH_3_OH) and BLI was performed 10 days after administration. Panel (**a**) shows representative images of an animal per group. The color bar indicates the relative bioluminescent signal intensities in photons/s/cm^2^/sr. (**b**) Quantification of the BLI signal expressed as the relative percentage of the saline group. The asterisk (*) indicates a significant difference as assessed by a two-tailed Student *t*-test for unpaired data (*p* < 0.05).

We tested the effects of methanol administration (1.5 g/kg i.p.) on cell proliferation in MITO-Luc mice. BLI imaging signals associated with cell proliferation decreased in MITO-Luc mice administered methanol in a similar fashion as observed upon ethanol administration, with an approximately 50% reduction 10 days after administration (Figure 4). This result further supports the suggestion that endogenously produced aldehydes have a detrimental effect on BM cell proliferation, which can be assessed as a decrement of bioluminescence in the MITO-Luc mouse model.

### 3.3. Proteome Analysis after Ethanol Administration Indicate Serum and Splenic Reduction of CXCL12 Levels

**Figure 5:**
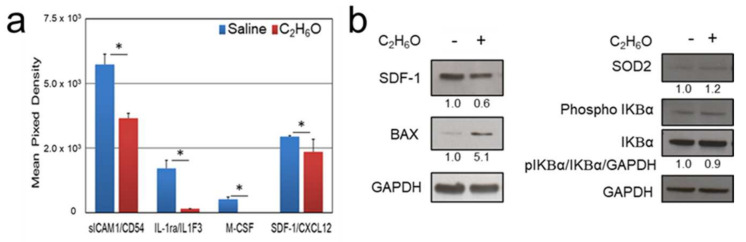
Effects of acute ethanol administration on cytokine/chemokine levels. (**a**) Proteome Profiler Mouse Cytokine Array (R&D Systems) analysis performed on pooled sera from MITO-Luc mice (N = 4 per group) 10 days after administration of either vehicle (saline) or ethanol solution. According to the manufacturer’s instructions, array data were quantified as mean pixel density, normalized to the density of the positive controls. (**b**) Immunoblot analysis performed on tissue homogenates from spleens collected from animals in the groups described above. Glyceraldehyde phosphate dehydrogenase (GAPDH) was used as a protein loading control. Band intensities were quantified using the ImageJ software and numbers indicate the normalized ratio of the indicated proteins to relative GAPDH signals.

Alcohol intoxication alters the plasma levels of a large panel of cytokines and growth factors [58,59], possibly affecting cell proliferation. In line with this evidence, using the Mouse Cytokine Array Panel, Proteome Profiler™ array (R&D Systems) we evaluated the serum levels of 40 cytokines and chemokines. Ten days after acute ethanol intake, in correspondence with the severe reduction of BLI emission in the spleen and the BM, we also determined a significant alteration of the circulating levels of soluble intercellular adhesion molecule-1 (sICAM-1), interleukin-1 receptor antagonist (IL-1ra/IL-1F3), macrophage colony-stimulating factor (M CSF), and stromal cell-derived factor-1 (SDF-1/CXCL12) compared to controls (Figure 5a). SDF-1 is a CXC chemokine family member constitutively expressed by BM stromal cells. Plasmatic reduction of the levels of SDF-1/CXCL12 has been described in patients with alcohol use disorders [60]. Considering its role in regulating HSC homeostasis and trafficking [61], we focused our attention on SDF-1/CXCL12, further confirming by immunoblot analysis the reduction of its expression in spleen homogenates collected from animals subjected to acute alcohol intoxication compared to controls (Figure 5b).

### 3.4. Immunoblot Analysis after Ethanol Administration Indicate Increased Splenic Expression of Oxidative Stress and Apoptosis Markers

To further examine the cellular mechanism that could underlie the relationship between alcohol exposure and reduced cell proliferation, we performed immunoblot analysis of the splenic tissue lysate obtained from animals sacrificed 10 days after administration of either saline or ethanol solution (Figure 5b). Acute ethanol-induced toxicity is associated with increased mitochondrial dysfunction and oxidative stress, triggering apoptosis. In response to alcohol intake-induced oxidative stress, mitochondrial manganese superoxide dismutase (MnSOD/SOD2) upregulation has been observed [62]. BCL2-associated X protein (BAX), a member of the pro-apoptotic B-cell lymphoma 2 family proteins, plays a pivotal role in the onset of apoptosis induced by ethanol in splenic T and B lymphocytes [63]. Acute and chronic alcohol intake may also induce changes in the levels of nuclear factor kappa light-chain enhancer of activated B cells (NF-κB), which regulate the immune response [64]. In ethanol-treated animals, we observed a moderate albeit significant increase of SOD2, consistent with its induction under oxidative stress conditions [48], and an increment of BAX expression, suggesting activation of the apoptotic signaling pathway [65]. These results are in agreement with previous reports describing apoptosis in splenocytes in the white pulp [66], splenic T and B cells [63,67], and macrophages [68] induced by alcohol administration. Conversely, in our experimental setting, NF-κB is not dysregulated as both the levels of total and phosphorylated inhibitory proteins of kB family (IkB) remained unaltered.

### 3.5. Short-Term Activation of NF-Y Driven Luciferase after Acute Ethanol Administration

**Figure 6:**
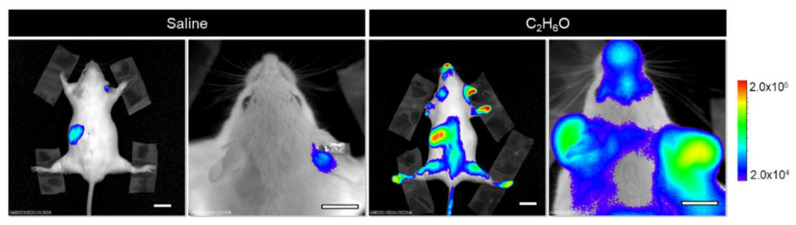
NF-Y-dependent luciferase activity in the MITO-Luc mouse model in response to acute ethanol administration. In vivo longitudinal bioluminescence imaging analysis of a representative animal 6 h after either saline or ethanol administration. The pseudo-color scale bar represents the relative bioluminescent signal intensities from the lowest (blue) to the highest (red); radiance is expressed in photons per second per square centimeter per steradian (photons/s/cm^2^/steradian).

Recently, NF-Y has been proven to participate in the early stages of alcoholic liver disease [69]; moreover, NF-Y, orchestrating the differential gene expression in response to acute alcohol intake, affects brain cell proliferation [70]. In particular, 6 h after ethanol (6 g/kg) administration in B6 and D2 mice, upregulation of NF-Y in the brain was determined by microarray analysis [70]. In line with these findings, 6 h after ethanol administration in MITO-Luc mice, we observed a widespread BLI signal also in the brain region, undetectable at baseline and in saline-treated controls (Figure 6).

## 4. Discussion

Alcohol is a leading risk factor for disease burden and premature mortality, as stated by the World Health Organization in the Global Status Report on Alcohol and Health [71]. Each year, 3.3 million deaths worldwide are attributable to alcohol consumption, with an expected increasing trend over the next decade [72]. A recent study reported an increase in binge drinking behavior among the general population [73]. In particular, binge drinking is commonly diffused among younger adults. Even if they may later develop alcohol-use problems, most young age binge drinkers are not alcoholics or alcohol dependent. They consider heavy episodic alcohol intake as a “rite of passage” and generally fail to recognize the associated risks of perturbation of the physiological and neurodevelopmental changes occurring in adolescence. Emerging evidence from studies involving young human subjects indicates that acute drinking is responsible for the following side effects: morphometric anomalies in different brain areas, as assessed by functional magnetic resonance imaging [74,75]; neuroimmune system impairment, leading to long-term psychological and behavioral dysfunctions [76]; deficits in memory and attention functions [77]; negative effects on proliferation and differentiation potential of different types of progenitor/stem cells [22]; and gut microbiota dysbiosis [78]. Alcohol intake is also associated with several conditions, including tumors; immunological disorders; cardiovascular diseases; impaired injury healing; mental and behavioral disorders; gastrointestinal conditions; lung, skeletal and muscular diseases; reproductive disorders; and pre-natal harm [79,80]. Moreover, there are additional social and economic burdens connected with alcohol drinking and addiction.

Excessive consumption of alcoholic beverages may have a severe impact on the immune system, leading to impairments in host defense, susceptibility to viral infection, reduced healing, and increased risk of developing tumors in several anatomical sites [23,81,82]. Approximately 5.5% of all cancer deaths (770,000 per year worldwide) are attributable to alcohol, with a loss of about 19 years of potential life for each victim [79,82,83]. Although the contribution of the precise cellular and molecular processes of alcohol-associated carcinogenesis has not been fully clarified, a casual role of the genotoxic effect of the ethanol metabolite acetaldehyde and its by-products, the DNA damage induced by the increased level of reactive oxygen species, and the direct or indirect alterations of oncogenic or regulatory pathways have been proposed [23,84,85,86,87]. In particular, impaired hematopoiesis has been linked to the genotoxic action on hematopoietic stem cells (HSCs) of reactive aldehydes produced during ethanol metabolism [43,88]. In primitive BM-derived HSCs, preferential expression of the nuclear transcription factor Y (NF-Y) dictates self-renewal, proliferation, and survival [89,90,91]. Microarray analysis has shown that ethanol exposure determines the modulation of genes involved in cell proliferation, growth arrest, apoptosis, and DNA damage in peripheral blood both in animal models and humans [92]. Interestingly, NF-Y regulates the expression of several ethanol-responsive genes in mice subjected to acute alcohol intoxication [70]. Moreover, acting as a regulator of major histocompatibility complex (MHC) gene expression [93] and macrophage maturation [94], NF-Y is involved in the immune response. Consistently, in the MITO-Luc mouse model, in which the luciferase gene expression is driven by the activity of a NF-Y-dependent cyclin B2 promoter, we observed a rapid increase in BM luciferase activity after acute ethanol intake. Interestingly, NF-Y-driven luciferase activity in hematopoietic organs was then reduced to baseline levels in approximately 4 weeks. This result is in agreement with a previous report describing that alcohol-dependent BM damage affecting hematopoiesis is reversible after abstinence for a month [41]. We previously reported that the activation of microglial cells induced by supraphysiological levels of unconjugated bilirubin can induce luciferase emission in the brain of MITO-Luc mice [28]. In line with these findings, we observed an increment in brain BLI emission 6 h after acute ethanol administration, consistent with possible proliferation of microglial cells, which has been described as one of the pathophysiological consequences of alcohol intake [95,96,97].

Along with ethanol as its major metabolic by-product, acetaldehyde is also considered as a carcinogen by the International Agency for Research on Cancer. As a matter of fact, ethanol, directly or through its metabolic intermediates, determines an impairment of neuronal [98,99], hepatic [100], and hematopoietic progenitor cell proliferation [22,101]. Specifically, the susceptibility of HSCs to acetaldehyde has been demonstrated [43] and toxicity on BM precursor cells was observed in heavy drinkers [102]. Both in mice [45] and humans [103], in the absence of the protective role of the Fanconi anemia DNA repair pathway and the activity of an isoform of aldehyde dehydrogenases (ALDH2), ethanol’s genotoxic effect results in severe depletion of the HSCs pool [104]. Harmful effects of in utero alcohol exposure on hematopoiesis in BM and spleen have been described in experimental models [105,106]. Furthermore, epidemiological studies demonstrated the association between maternal alcohol consumption during pregnancy and development of childhood leukemia, suggesting a possible toxic role of ethanol and/or its metabolites on fetal bone marrow hematopoiesis [107]. Moreover, acetaldehyde produced during ethanol oxidation mediates G2/M cell cycle arrest [108]. Interestingly, about 1–2% of nucleated BM cells, including hematopoietic, mesenchymal, and endothelial precursors, express high levels of ALDH [109], implicated in the regulation of cell proliferation and resistance to exogenous stress [110]. In particular, ALDH modulation affects HSCs’ self-renewal via inhibition of retinoic acid signaling [111]. We used our model to evaluate the response to pharmacological modulation of ALDH as a potential therapeutic target to combat aldehyde toxicity [112]. Understanding the mechanisms of in vivo aldehyde production and de-toxification is particularly relevant considering that more than 560 million people in the world, being characterized by the presence of the ALDH2*2 genetic variants [113,114], are more susceptible to aldehyde toxicity. Indeed, we determined that ALDH activation restored, at least in part, physiological cell proliferation upon alcohol intake, while inhibition of ALDH activity had a detrimental effect.

Overall, our experimental data indicate that acute alcohol administration affects cell proliferation in the bone marrow and spleen. The study was performed on a transgenic animal model, which offers unique opportunities to dissect in vivo the pathophysiological effects of alcohol on cell proliferation. Caution should be exercised in translating information obtained from alcohol studies performed on rodents into clinical treatments in humans since no animal model completely reproduces all the complex aspects of human alcohol consumption and intoxication [26]. Nonetheless, the results are broadly consistent with clinical data observed in alcoholic patients indicating that alcohol and its metabolite acetaldehyde exert a cytotoxic effect on hematopoietic cells [21,55] and further support the notion that ALDH might represent a druggable target for protection of alcohol and aldehyde toxicity.

## 5. Conclusions

Alcohol intake determines a transient modulation in the bioluminescence emission in the MITO-Luc mouse, where the light intensity is associated with cellular proliferative status. Activation of ALDH activity and administration of an antioxidant compound partially protected the animals from a reduction of cell proliferation in BM and spleen; conversely, inhibition of ALDH activity had a detrimental effect. Collectively, these data are in agreement with a previously established impairment on the antitumor immune response associated with the cytotoxic effect on HSCs exerted by aldehydes produced by both ethanol and methanol metabolism. The proposed experimental model might be instrumental for further in vivo elucidation of the mechanisms of aldehydes’ endogenous production and de-toxification.

## Data Availability

The data generated during the current study are available from the corresponding author upon reasonable request.

## Supplementary Materials

The following are available online at https://www.mdpi.com/article/10.3390/cancers13194999/s1, Figure S1: Uncropped WB.

## References

[1] Pflaum T., Hausler T., Baumung C., Ackermann S., Kuballa T., Rehm J., Lachenmeier D.W. (2016). Carcinogenic compounds in alcoholic beverages: An update. Arch. Toxicol..

[2] Szabo G., Mandrekar P. (2009). A recent perspective on alcohol, immunity, and host defense. Alcohol Clin. Exp. Res..

[3] Blot W.J. (1992). Alcohol and cancer. Cancer Res..

[4] Jung M.K., Callaci J.J., Lauing K.L., Otis J.S., Radek K.A., Jones M.K., Kovacs E.J. (2011). Alcohol exposure and mechanisms of tissue injury and repair. Alcohol Clin. Exp. Res..

[5] Molina P.E., Happel K.I., Zhang P., Kolls J.K., Nelson S. (2010). Focus on: Alcohol and the immune system. Alcohol Res. Health.

[6] Messingham K.A., Faunce D.E., Kovacs E.J. (2002). Alcohol, injury, and cellular immunity. Alcohol.

[7] Brown L.A., Cook R.T., Jerrells T.R., Kolls J.K., Nagy L.E., Szabo G., Wands J.R., Kovacs E.J. (2006). Acute and chronic alcohol abuse modulate immunity. Alcohol Clin. Exp. Res..

[8] Yen C.H., Ho P.S., Yeh Y.W., Liang C.S., Kuo S.C., Huang C.C., Chen C.Y., Shih M.C., Ma K.H., Sung Y.F. (2017). Differential cytokine levels between early withdrawal and remission states in patients with alcohol dependence. Psychoneuroendocrinology.

[9] Barr T., Helms C., Grant K., Messaoudi I. (2016). Opposing effects of alcohol on the immune system. Prog. Neuropsychopharmacol. Biol. Psychiatry.

[10] Thinnahanumaih M., Puranik N., Bidare C., Kammar K.F., Venkatakrishniah O.K., Maitri V. (2012). Moderate alcohol intake even a short duration has deleterious effects on hematologic profile in Indian men. Int. J. Med. Sci. Public Health.

[11] Gauthier T.W. (2015). Prenatal alcohol exposure and the developing immune system. Alcohol Res..

[12] Lindenbaum J. (1987). Hematologic complications of alcohol abuse. Semin Liver Dis.

[13] Zhang H., Zhu Z., Zhang F., Meadows G.G. (2015). Alcohol consumption and antitumor immunity: Dynamic changes from activation to accelerated deterioration of the immune system. Adv. Exp. Med. Biol..

[14] Scherübl H. (2020). Alcohol use and gastrointestinal cancer risk. Visc. Med..

[15] Ballard H.S. (1997). The hematological complications of alcoholism. Alcohol Health Res. World.

[16] Díaz L.E., Montero A., González-Gross M., Vallejo A.I., Romeo J., Marcos A. (2002). Influence of alcohol consumption on immunological status: A review. Eur. J. Clin. Nutr..

[17] Pasala S., Barr T., Messaoudi I. (2015). Impact of alcohol abuse on the adaptive immune system. Alcohol Res..

[18] Fröbel J., Landspersky T., Percin G., Schreck C., Rahmig S., Ori A., Nowak D., Essers M., Waskow C., Oostendorp R.A.J. (2021). The hematopoietic bone marrow niche ecosystem. Front. Cell Dev. Biol..

[19] Huff N.K., Spencer N.D., Gimble J.M., Bagby G.J., Nelson S., Lopez M.J. (2011). Impaired expansion and multipotentiality of adult stromal cells in a rat chronic alcohol abuse model. Alcohol.

[20] Taffe M.A., Kotzebue R.W., Crean R.D., Crawford E.F., Edwards S., Mandyam C.D. (2010). Long-lasting reduction in hippocampal neurogenesis by alcohol consumption in adolescent nonhuman primates. Proc. Natl. Acad. Sci. USA.

[21] Meagher R.C., Sieber F., Spivak J.L. (1982). Suppression of hematopoietic-progenitor-cell proliferation by ethanol and acetaldehyde. N. Engl. J. Med..

[22] Di Rocco G., Baldari S., Pani G., Toietta G. (2019). Stem cells under the influence of alcohol: Effects of ethanol consumption on stem/progenitor cells. Cell. Mol. Life Sci..

[23] Boffetta P., Hashibe M. (2006). Alcohol and cancer. Lancet Oncol..

[24] Hao H.N., Parker G.C., Zhao J., Barami K., Lyman W.D. (2003). Differential responses of human neural and hematopoietic stem cells to ethanol exposure. J. Hematother. Stem Cell Res..

[25] Varlamov O., Bucher M., Myatt L., Newman N., Grant K.A. (2020). Daily ethanol drinking followed by an abstinence period impairs bone marrow niche and mitochondrial function of hematopoietic stem/progenitor cells in rhesus macaques. Alcohol Clin. Exp. Res..

[26] Crabbe J.C., Phillips T.J., Belknap J.K. (2010). The complexity of alcohol drinking: Studies in rodent genetic models. Behav. Genet..

[27] Goeman F., Manni I., Artuso S., Ramachandran B., Toietta G., Bossi G., Rando G., Cencioni C., Germoni S., Straino S. (2012). Molecular imaging of nuclear factor-Y transcriptional activity maps proliferation sites in live animals. Mol. Biol. Cell.

[28] Manni I., Di Rocco G., Fusco S., Leone L., Barbati S.A., Carapella C.M., Grassi C., Piaggio G., Toietta G. (2017). Monitoring the response of hyperbilirubinemia in the mouse brain by in vivo bioluminescence imaging. Int. J. Mol. Sci..

[29] Rizzi N., Manni I., Vantaggiato C., Delledonne G.A., Gentileschi M.P., Maggi A., Piaggio G., Ciana P. (2015). In vivo imaging of cell proliferation for a dynamic, whole body, analysis of undesired drug effects. Toxicol. Sci..

[30] Courties G., Herisson F., Sager H.B., Heidt T., Ye Y., Wei Y., Sun Y., Severe N., Dutta P., Scharff J. (2015). Ischemic stroke activates hematopoietic bone marrow stem cells. Circ. Res..

[31] Carpenter R.S., Marbourg J.M., Brennan F.H., Mifflin K.A., Hall J.C.E., Jiang R.R., Mo X.M., Karunasiri M., Burke M.H., Dorrance A.M. (2020). Spinal cord injury causes chronic bone marrow failure. Nat. Commun..

[32] Goral J., Karavitis J., Kovacs E.J. (2008). Exposure-dependent effects of ethanol on the innate immune system. Alcohol.

[33] Zhong W., Zhang W., Li Q., Xie G., Sun Q., Sun X., Tan X., Jia W., Zhou Z. (2015). Pharmacological activation of aldehyde dehydrogenase 2 by Alda-1 reverses alcohol-induced hepatic steatosis and cell death in mice. J. Hepatol..

[34] Hillbom M.E., Sarviharju M.S., Lindros K.O. (1983). Potentiation of ethanol toxicity by cyanamide in relation to acetaldehyde accumulation. Toxicol. Appl. Pharmacol.

[35] Carito V., Ceccanti M., Cestari V., Natella F., Bello C., Coccurello R., Mancinelli R., Fiore M. (2017). Olive polyphenol effects in a mouse model of chronic ethanol addiction. Nutrition.

[36] Ward K.W., Perkins R.A., Kawagoe J.L., Pollack G.M. (1995). Comparative toxicokinetics of methanol in the female mouse and rat. Fundam. Appl. Toxicol..

[37] Baldari S., Di Rocco G., Trivisonno A., Samengo D., Pani G., Toietta G. (2016). Promotion of survival and engraftment of transplanted adipose tissue-derived stromal and vascular cells by overexpression of manganese superoxide dismutase. Int. J. Mol. Sci..

[38] Di Rocco G., Gentile A., Antonini A., Truffa S., Piaggio G., Capogrossi M., Toietta G. (2012). Analysis of biodistribution and engraftment into the liver of genetically modified mesenchymal stromal cells derived from adipose tissue. Cell Transpl..

[39] Wands J.R., Carter E.A., Bucher N.L., Isselbacher K.J. (1979). Inhibition of hepatic regeneration in rats by acute and chronic ethanol intoxication. Gastroenterology.

[40] Smith C., Gasparetto M., Jordan C., Pollyea D.A., Vasiliou V. (2015). The effects of alcohol and aldehyde dehydrogenases on disorders of hematopoiesis. Adv. Exp. Med. Biol..

[41] Casagrande G., Michot F. (1989). Alcohol-induced bone marrow damage: Status before and after a 4-week period of abstinence from alcohol with or without disulfiram. A randomized bone marrow study in alcohol-dependent individuals. Blut.

[42] Guo R., Ren J. (2010). Alcohol and acetaldehyde in public health: From marvel to menace. Int. J. Environ. Res. Public Health.

[43] Garaycoechea J.I., Crossan G.P., Langevin F., Daly M., Arends M.J., Patel K.J. (2012). Genotoxic consequences of endogenous aldehydes on mouse haematopoietic stem cell function. Nature.

[44] Joenje H. (2011). Metabolism: Alcohol, DNA and disease. Nature.

[45] Langevin F., Crossan G.P., Rosado I.V., Arends M.J., Patel K.J. (2011). Fancd2 counteracts the toxic effects of naturally produced aldehydes in mice. Nature.

[46] Jamal M., Ameno K., Tanaka N., Ito A., Takakura A., Kumihashi M., Kinoshita H. (2016). Ethanol and acetaldehyde after intraperitoneal administration to Aldh2-knockout mice-reflection in blood and brain levels. Neurochem. Res..

[47] Das S.K., Vasudevan D.M. (2007). Alcohol-induced oxidative stress. Life Sci..

[48] Koch O.R., Pani G., Borrello S., Colavitti R., Cravero A., Farrè S., Galeotti T. (2004). Oxidative stress and antioxidant defenses in ethanol-induced cell injury. Mol. Aspects Med..

[49] Koop D.R. (2006). Alcohol metabolism’s damaging effects on the cell: A focus on reactive oxygen generation by the enzyme cytochrome P450 2E1. Alcohol Res. Health.

[50] Brocardo P.S., Gil-Mohapel J., Christie B.R. (2011). The role of oxidative stress in fetal alcohol spectrum disorders. Brain Res. Rev..

[51] Arteel G.E. (2003). Oxidants and antioxidants in alcohol-induced liver disease. Gastroenterology.

[52] Joya X., Garcia-Algar O., Salat-Batlle J., Pujades C., Vall O. (2015). Advances in the development of novel antioxidant therapies as an approach for fetal alcohol syndrome prevention. Birth Defects Res. A Clin. Mol. Teratol..

[53] De Nicoló S., Tarani L., Ceccanti M., Maldini M., Natella F., Vania A., Chaldakov G.N., Fiore M. (2013). Effects of olive polyphenols administration on nerve growth factor and brain-derived neurotrophic factor in the mouse brain. Nutrition.

[54] Alirezaei M., Dezfoulian O., Neamati S., Rashidipour M., Tanideh N., Kheradmand A. (2012). Oleuropein prevents ethanol-induced gastric ulcers via elevation of antioxidant enzyme activities in rats. J. Physiol. Biochem..

[55] Pontel L.B., Rosado I.V., Burgos-Barragan G., Garaycoechea J.I., Yu R., Arends M.J., Chandrasekaran G., Broecker V., Wei W., Liu L. (2015). Endogenous formaldehyde is a hematopoietic stem cell genotoxin and metabolic carcinogen. Mol. Cell.

[56] Wei C., Wen H., Yuan L., McHale C.M., Li H., Wang K., Yuan J., Yang X., Zhang L. (2017). Formaldehyde induces toxicity in mouse bone marrow and hematopoietic stem/progenitor cells and enhances benzene-induced adverse effects. Arch. Toxicol..

[57] Szende B., Tyihák E. (2010). Effect of formaldehyde on cell proliferation and death. Cell Biol. Int..

[58] Achur R.N., Freeman W.M., Vrana K.E. (2010). Circulating cytokines as biomarkers of alcohol abuse and alcoholism. J. Neuroimmune Pharmacol..

[59] Crews F.T., Bechara R., Brown L.A., Guidot D.M., Mandrekar P., Oak S., Qin L., Szabo G., Wheeler M., Zou J. (2006). Cytokines and alcohol. Alcohol Clin. Exp. Res..

[60] García-Marchena N., Araos P.F., Barrios V., Sánchez-Marín L., Chowen J.A., Pedraz M., Castilla-Ortega E., Romero-Sanchiz P., Ponce G., Gavito A.L. (2016). Plasma chemokines in patients with alcohol use disorders: Association of CCL11 (Eotaxin-1) with psychiatric comorbidity. Front. Psychiatry.

[61] Sugiyama T., Kohara H., Noda M., Nagasawa T. (2006). Maintenance of the hematopoietic stem cell pool by CXCL12-CXCR4 chemokine signaling in bone marrow stromal cell niches. Immunity.

[62] Koch O.R., De Leo M.E., Borrello S., Palombini G., Galeotti T. (1994). Ethanol treatment up-regulates the expression of mitochondrial manganese superoxide dismutase in rat liver. Biochem. Biophys. Res. Commun..

[63] Slukvin I.I., Jerrells T.R. (1995). Different pathways of in vitro ethanol-induced apoptosis in thymocytes and splenic T and B lymphocytes. Immunopharmacology.

[64] Nowak A.J., Relja B. (2020). The impact of acute or chronic alcohol intake on the NF-κB signaling pathway in alcohol-related liver disease. Int. J. Mol. Sci..

[65] von Haefen C., Sifringer M., Menk M., Spies C.D. (2011). Ethanol enhances susceptibility to apoptotic cell death via down-regulation of autophagy-related proteins. Alcohol Clin. Exp. Res..

[66] Eid N.A., Ito Y., Li Z., Abe H., Kusakabe K., Shibata M.A., Otsuki Y. (2000). The relationship between apoptosis and splenocyte depletion in rats following ethanol treatment. Med. Electron. Microsc..

[67] Saad A.J., Jerrells T.R. (1991). Flow cytometric and immunohistochemical evaluation of ethanol-induced changes in splenic and thymic lymphoid cell populations. Alcohol Clin. Exp. Res..

[68] Singhal P.C., Reddy K., Ding G., Kapasi A., Franki N., Ranjan R., Nwakoby I.E., Gibbons N. (1999). Ethanol-induced macrophage apoptosis: The role of TGF-beta. J. Immunol..

[69] Zhang Y., Sun Y., Miao Q., Wang Q., Yang B., Li Y., Li L., Zhang R. (2021). Nuclear factor Y participates in alcoholic liver disease by activating SREBP1 expression in mice. Biochem. Biophys. Res. Commun..

[70] Uddin R.K., Singh S.M. (2006). cis-Regulatory sequences of the genes involved in apoptosis, cell growth, and proliferation may provide a target for some of the effects of acute ethanol exposure. Brain Res..

[71] Poznyak V., Rekve D. Global Status Report on Alcohol and Health 2018. https://apps.who.int/iris/handle/10665/274603.

[72] Manthey J., Shield K.D., Rylett M., Hasan O.S.M., Probst C., Rehm J. (2019). Global alcohol exposure between 1990 and 2017 and forecasts until 2030: A modelling study. Lancet.

[73] Azagba S., Shan L., Latham K., Manzione L. (2020). Trends in binge and heavy drinking among adults in the United States, 2011–2017. Subst. Use Misuse.

[74] Cservenka A., Brumback T. (2017). The burden of binge and heavy drinking on the brain: Effects on adolescent and young adult neural structure and function. Front. Psychol..

[75] Jones S.A., Lueras J.M., Nagel B.J. (2018). Effects of binge drinking on the developing brain. Alcohol Res..

[76] Pascual M., Montesinos J., Guerri C. (2018). Role of the innate immune system in the neuropathological consequences induced by adolescent binge drinking. J. Neurosci. Res..

[77] Hermens D.F., Lagopoulos J. (2018). Binge drinking and the young brain: A mini review of the neurobiological underpinnings of alcohol-induced blackout. Front. Psychol..

[78] Lee E., Lee J.-E. (2021). Impact of drinking alcohol on gut microbiota: Recent perspectives on ethanol and alcoholic beverage. Curr. Opin. Food Sci..

[79] Praud D., Rota M., Rehm J., Shield K., Zatoński W., Hashibe M., La Vecchia C., Boffetta P. (2016). Cancer incidence and mortality attributable to alcohol consumption. Int. J. Cancer.

[80] Rehm J., Gmel G.E., Gmel G., Hasan O.S.M., Imtiaz S., Popova S., Probst C., Roerecke M., Room R., Samokhvalov A.V. (2017). The relationship between different dimensions of alcohol use and the burden of disease—An update. Addiction.

[81] Parkin D.M. (2011). Cancers attributable to consumption of alcohol in the UK in 2010. Br. J. Cancer.

[82] Nelson D.E., Jarman D.W., Rehm J., Greenfield T.K., Rey G., Kerr W.C., Miller P., Shield K.D., Ye Y., Naimi T.S. (2013). Alcohol-attributable cancer deaths and years of potential life lost in the United States. Am. J. Public Health.

[83] Rumgay H., Shield K., Charvat H., Ferrari P., Sornpaisarn B., Obot I., Islami F., Lemmens V.E.P.P., Rehm J., Soerjomataram I. (2021). Global burden of cancer in 2020 attributable to alcohol consumption: A population-based study. Lancet Oncol..

[84] Ratna A., Mandrekar P. (2017). Alcohol and cancer: Mechanisms and therapies. Biomolecules.

[85] Nieminen M.T., Salaspuro M. (2018). Local acetaldehyde—An essential role in alcohol-related upper gastrointestinal tract carcinogenesis. Cancers.

[86] Stornetta A., Guidolin V., Balbo S. (2018). Alcohol-derived acetaldehyde exposure in the oral cavity. Cancers.

[87] Rossi M., Jahanzaib Anwar M., Usman A., Keshavarzian A., Bishehsari F. (2018). Colorectal cancer and alcohol consumption-populations to molecules. Cancers.

[88] Milsom M.D. (2018). Stem cells spirited away by alcohol-induced DNA damage. Hemasphere.

[89] Bungartz G., Land H., Scadden D.T., Emerson S.G. (2012). NF-Y is necessary for hematopoietic stem cell proliferation and survival. Blood.

[90] Radomska H.S., Satterthwaite A.B., Taranenko N., Narravula S., Krause D.S., Tenen D.G. (1999). A nuclear factor Y (NFY) site positively regulates the human CD34 stem cell gene. Blood.

[91] Zhu J., Zhang Y., Joe G.J., Pompetti R., Emerson S.G. (2005). NF-Ya activates multiple hematopoietic stem cell (HSC) regulatory genes and promotes HSC self-renewal. Proc. Natl. Acad. Sci. USA.

[92] Hicks S.D., Lewis L., Ritchie J., Burke P., Abdul-Malak Y., Adackapara N., Canfield K., Shwarts E., Gentile K., Meszaros Z.S. (2012). Evaluation of cell proliferation, apoptosis, and DNA-repair genes as potential biomarkers for ethanol-induced CNS alterations. BMC Neurosci..

[93] Sachini N., Papamatheakis J. (2017). NF-Y and the immune response: Dissecting the complex regulation of MHC genes. Biochim Biophys. Acta Gene Regul. Mech..

[94] Valledor A.F., Borràs F.E., Cullell-Young M., Celada A. (1998). Transcription factors that regulate monocyte/macrophage differentiation. J. Leukoc. Biol..

[95] He J., Crews F.T. (2008). Increased MCP-1 and microglia in various regions of the human alcoholic brain. Exp. Neurol..

[96] Marshall S.A., Geil C.R., Nixon K. (2016). Prior binge ethanol exposure potentiates the microglial response in a model of alcohol-induced neurodegeneration. Brain Sci..

[97] McClain J.A., Morris S.A., Deeny M.A., Marshall S.A., Hayes D.M., Kiser Z.M., Nixon K. (2011). Adolescent binge alcohol exposure induces long-lasting partial activation of microglia. Brain Behav. Immun..

[98] Nixon K., Crews F.T. (2002). Binge ethanol exposure decreases neurogenesis in adult rat hippocampus. J. Neurochem..

[99] Sutherland G.T., Sheahan P.J., Matthews J., Dennis C.V., Sheedy D.S., McCrossin T., Curtis M.A., Kril J.J. (2013). The effects of chronic alcoholism on cell proliferation in the human brain. Exp. Neurol..

[100] Shi X., Chang C.C., Basson M.D., Upham B.L., Wei L., Zhang P. (2014). Alcohol disrupts human liver stem/progenitor cell proliferation and differentiation. J. Stem Cell Res. Ther..

[101] Wang H., Zhou H., Chervenak R., Moscatello K.M., Brunson L.E., Chervenak D.C., Wolcott R.M. (2009). Ethanol exhibits specificity in its effects on differentiation of hematopoietic progenitors. Cell Immunol..

[102] Latvala J., Parkkila S., Melkko J., Niemelä O. (2001). Acetaldehyde adducts in blood and bone marrow of patients with ethanol-induced erythrocyte abnormalities. Mol. Med..

[103] Hira A., Yabe H., Yoshida K., Okuno Y., Shiraishi Y., Chiba K., Tanaka H., Miyano S., Nakamura J., Kojima S. (2013). Variant ALDH2 is associated with accelerated progression of bone marrow failure in Japanese Fanconi anemia patients. Blood.

[104] Van Wassenhove L.D., Mochly-Rosen D., Weinberg K.I. (2016). Aldehyde dehydrogenase 2 in aplastic anemia, Fanconi anemia and hematopoietic stem cells. Mol. Genet. Metab..

[105] Wang H., Zhou H., Moscatello K.M., Dixon C., Brunson L.E., Chervenak R., Chervenak D.C., Zhao X., Wolcott R.M. (2006). In utero exposure to alcohol alters cell fate decisions by hematopoietic progenitors in the bone marrow of offspring mice during neonatal development. Cell Immunol..

[106] Moscatello K.M., Biber K.L., Jennings S.R., Chervenak R., Wolcott R.M. (1999). Effects of in utero alcohol exposure on B cell development in neonatal spleen and bone marrow. Cell Immunol..

[107] Latino-Martel P., Chan D.S., Druesne-Pecollo N., Barrandon E., Hercberg S., Norat T. (2010). Maternal alcohol consumption during pregnancy and risk of childhood leukemia: Systematic review and meta-analysis. Cancer Epidemiol. Biomark. Prev..

[108] Scheer M.A., Schneider K.J., Finnigan R.L., Maloney E.P., Wells M.A., Clemens D.L. (2016). The involvement of acetaldehyde in ethanol-induced cell cycle impairment. Biomolecules.

[109] Gentry T., Foster S., Winstead L., Deibert E., Fiordalisi M., Balber A. (2007). Simultaneous isolation of human BM hematopoietic, endothelial and mesenchymal progenitor cells by flow sorting based on aldehyde dehydrogenase activity: Implications for cell therapy. Cytotherapy.

[110] Vassalli G. (2019). Aldehyde dehydrogenases: Not just markers, but functional regulators of stem cells. Stem Cells Int..

[111] Chute J.P., Muramoto G.G., Whitesides J., Colvin M., Safi R., Chao N.J., McDonnell D.P. (2006). Inhibition of aldehyde dehydrogenase and retinoid signaling induces the expansion of human hematopoietic stem cells. Proc. Natl. Acad. Sci. USA.

[112] Dollé L., Gao B. (2015). Pharmacological chaperone therapies: Can aldehyde dehydrogenase activator make us healthier?. J. Hepatol..

[113] Wang W., Wang C., Xu H., Gao Y. (2020). Aldehyde dehydrogenase, liver disease and cancer. Int. J. Biol. Sci..

[114] Chen C.H., Ferreira J.C.B., Joshi A.U., Stevens M.C., Li S.J., Hsu J.H., Maclean R., Ferreira N.D., Cervantes P.R., Martinez D.D. (2020). Novel and prevalent non-East Asian ALDH2 variants; Implications for global susceptibility to aldehydes’ toxicity. EBioMedicine.

