# Supplementary figures and images for: Reduction of Cell Proliferation by Acute C2H6O Exposure
Source: Cancers (Basel). 2021 Oct 5;13(19):4999. doi: 10.3390/cancers13194999 (PMC8508324; doi:10.3390/cancers13194999)

B

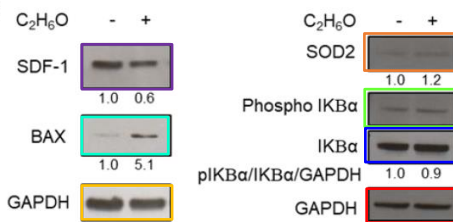

ETOH E MARKER APOPTOSICI 4-12% e 12%

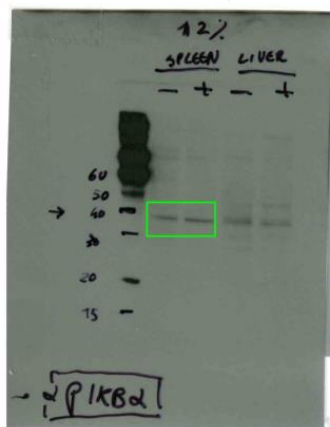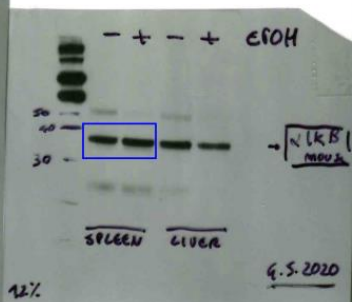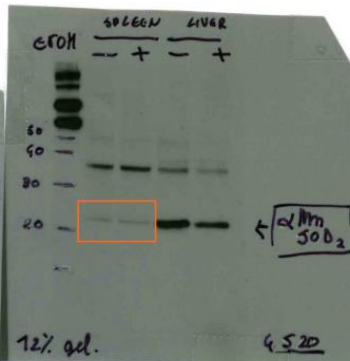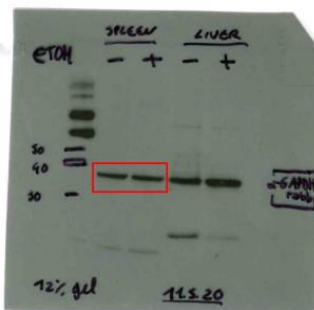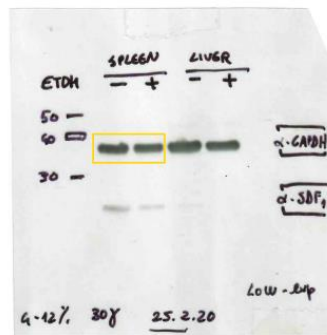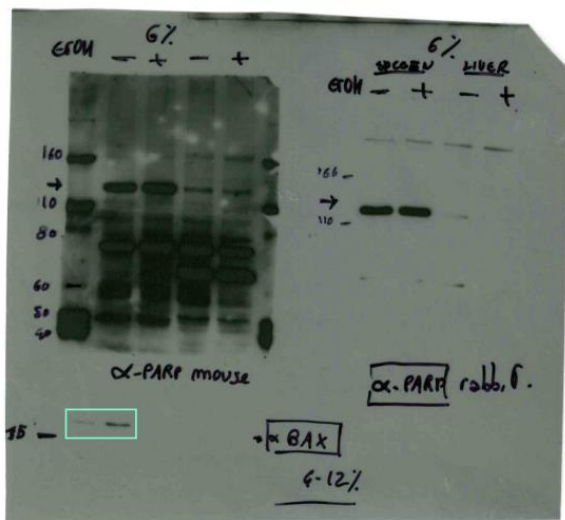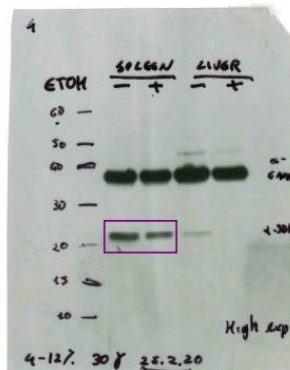

Supplement: Supplementary file 1 [file cancers-13-04999-s001.zip › cancers-1367562-supplementary.pdf]
